# Supplementary material for: Role of Soluble ST2 Biomarker in Predicting Recurrence of Atrial Fibrillation after Electrical Cardioversion or Pulmonary Vein Isolation
Source: Int J Mol Sci. 2023 Sep 13;24(18):14045. doi: 10.3390/ijms241814045 (PMC10531224; doi:10.3390/ijms241814045)
Supplement: Supplementary file 1 [file ijms-24-14045-s001.zip › ijms-2568377-supplementary.pdf]

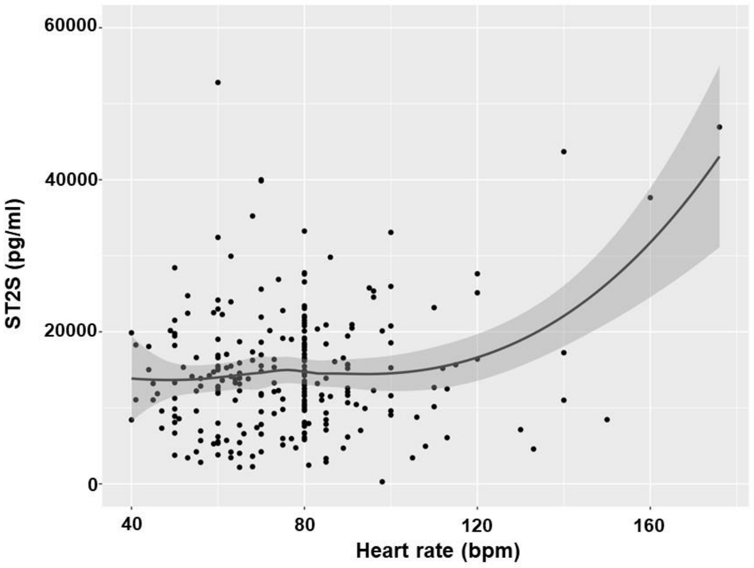

Figure S1. Supplementary material. Baseline sST2 and ventricular rate.

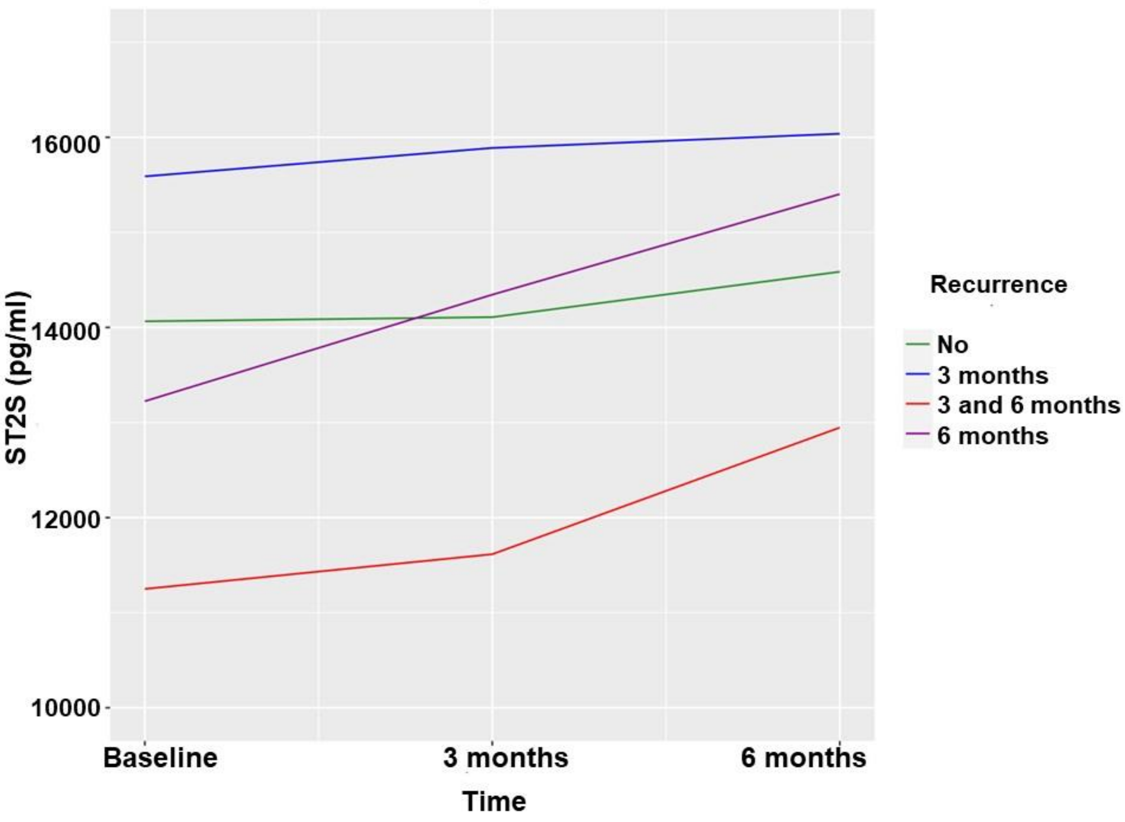

Figure S2. Supplementary material. sST2 and atrial fibrillation recurrence in persistent AF patients.

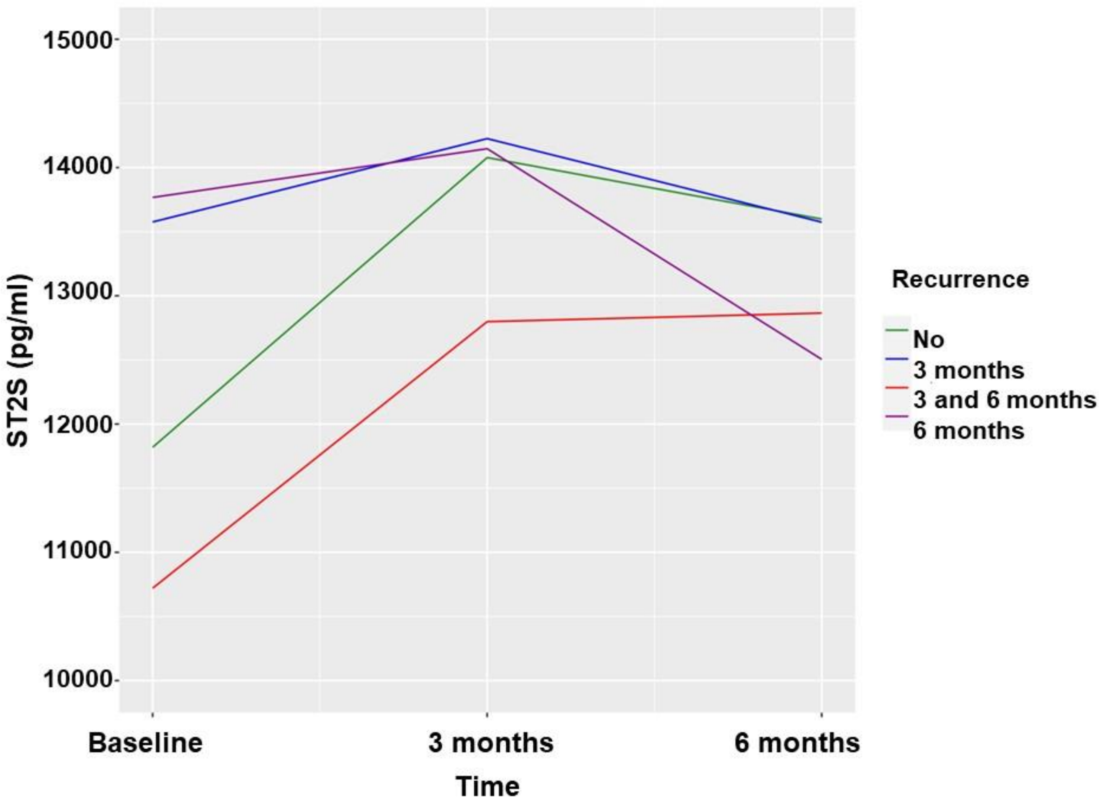

Figure S3. Supplementary material. sST2 and AF recurrence in paroxysmal AF patients.

Table S1 suplementary material. Differences in the sST2 biomarker in AF recurrence at follow-up of patients with persistent AF in the cohort of patients undergoing PVI.

3 months-FU		**sST2.0**	**sST2.1**	**p**
	No recurrence (n=65)	13,973 ± 8124	14,133 ± 6875	0.834
	Recurrence (n=23)	14,079 ± 7022	14,401 ± 7240	0.746
6 months-FU		**sST2.0**	**sST2.2**	**p**
	No recurrence (n=73)	14,377 ± 8306	14,883 ± 6878	0.401
	Recurrence (n=15)	12,170 ± 4485	14,092 ± 4370	0.183

sST2.0: sST2 at baseline; sST2.1: sST2 at 3 months-follow-up; sST2.2: sST2 at 6 months-follow-up.

Table S2 supplementary material. Differences in the sST2 biomarker in AF recurrence at follow-up of patients with paroxysmal AF in the cohort of patients undergoing PVI.

3 months FU		**sST2.0**	**sST2.1**	**p**
	No recurrence (n=60)	12,013 ± 6459	14,084 ± 9320	0.012*
	Recurrence (n=8)	12,861 ± 4989	13,868 ± 3496	0.603
6 months FU		**sST2.0**	**sST2.2**	**p**
	No recurrence (n=60)	11,994 ± 6016	13,595 ± 7018	0.012*
	Recurrence (n=8)	13,005 ± 8438	12,594 ± 4194	0.906

sST2.0: sST2 at baseline; sST2.1: sST2 at 3 months-FU; sST2.2: sST2 at 6 months-FU; FU: follow-up. *p<0.05

Table S3 supplementary material. Differences in the sST2 biomarker in patients who underwent ECV, excluding patients who underwent any follow-up intervention.

3 months-FU	Recurrence	**sST2.0**	**sST2.1**	**p**
	No (n=47)	18,598 ± 10916	14680 ± 7561	0.002*
	Yes (n=30)	14,674 ± 6199	15587 ± 7176	0.360
6 months-FU		**sST2.0**	**sST2.2**	**p**
	No (n=42)	16,298 ± 10,832	13952,5 ±7603,7	0.05
	Yes (n=35)	17,994 ± 7693	18454,6 ±9453,6	0.727
3 vs 6 months-FU		**ST2S.1**	**ST2S.2**	**p**
	No (n=42)	13,025 ± 6301	13,952 ± 7603	0.143
	Yes (n=35)	17,444 ± 7929	18,454 ± 9453	0.221

Values expressed in pg/mL, as mean ± standard deviation. sST2.0: sST2 at basal, sST2.1: sST2 at 3 months-FU. sST2.2: sST2 at 6 months-FU. AF: atrial fibrillation; ECV: Electrical cardioversion.

*p<0.05
